# Implementing clinical guidelines in psychiatry: a qualitative study of perceived facilitators and barriers

**DOI:** 10.1186/1471-244X-10-8

**Published:** 2010-01-20

**Authors:** Tord Forsner, Johan Hansson, Mats Brommels, Anna Åberg Wistedt, Yvonne Forsell

**Affiliations:** 1 Department of Public Health Sciences, Karolinska Institutet, Stockholm SE-171 76, Sweden; 2 Medical Management Centre, Department of Learning, Informatics, Management, and Ethics, Karolinska Institutet, Stockholm SE-171 77, Sweden; 3 Department of Public Health, University of Helsinki, Helsinki FIN-00014, Finland; 4 Department of Clinical Neuroscience, Section of Psychiatry St Göran's Hospital, Karolinska Institutet, Stockholm SE-112 81, Sweden

## Abstract

**Background:**

Translating scientific evidence into daily practice is complex. Clinical guidelines can improve health care delivery, but there are a number of challenges in guideline adoption and implementation. Factors influencing the effective implementation of guidelines remain poorly understood. Understanding of barriers and facilitators is important for development of effective implementation strategies. The aim of this study was to determine perceived facilitators and barriers to guideline implementation and clinical compliance to guidelines for depression in psychiatric care.

**Methods:**

This qualitative study was conducted at two psychiatric clinics in Stockholm, Sweden. The implementation activities at one of the clinics included local implementation teams, seminars, regular feedback and academic detailing. The other clinic served as a control and only received guidelines by post. Data were collected from three focus groups and 28 individual, semi-structured interviews. Content analysis was used to identify themes emerging from the interview data.

**Results:**

The identified barriers to, and facilitators of, the implementation of guidelines could be classified into three major categories: (1) organizational resources, (2) health care professionals' individual characteristics and (3) perception of guidelines and implementation strategies. The practitioners in the implementation team and at control clinics differed in three main areas: (1) concerns about control over professional practice, (2) beliefs about evidence-based practice and (3) suspicions about financial motives for guideline introduction.

**Conclusions:**

Identifying the barriers to, and facilitators of, the adoption of recommendations is an important way of achieving efficient implementation strategies. The findings of this study suggest that the adoption of guidelines may be improved if local health professionals actively participate in an ongoing implementation process and identify efficient strategies to overcome barriers on an organizational and individual level. Getting evidence into practice and implementing clinical guidelines are dependent upon more than practitioners' motivation. There are factors in the local context, e.g. culture and leadership, evaluation, feedback on performance and facilitation, -that are likely to be equally influential.

## Background

Only approximately half of the patients visiting general medical practitioners receive treatment which differs from recommended best practice [1]. In psychiatry the number is unknown due to a lack of studies. Efficient strategies need to be developed that address barriers to the implementation of new knowledge and findings from research. However, the challenges of implementing evidence-based practice are complex and widespread.

Interest in clinical guidelines as an instrument to implement new knowledge and research findings has increased over the past decade [2]. Clinical guidelines are "systematically developed statements to assist practitioners and patient decisions about appropriate health care for specific clinical circumstances" [3], and are often used tools for promoting evidence-based practice [4]. They may lead to improved quality of care by decreasing inappropriate variation in clinical practice and ensuring that recent advances in medical knowledge are disseminated rapidly into everyday clinical practice [5]. Increasing efforts are being undertaken to transfer guidelines into clinical practice [6], but many attitudinal and behavioural barriers prevent physicians from adopting them [5]. Consequently, it remains uncertain how these clinical guidelines can best be implemented and used in clinical settings [7].

There is a growing literature that explores the barriers to the implementation of clinical guidelines in health care, and that identifies effective strategies for translating research into practice [2,8]. Regarding general medical practice, ineffective interventions include traditional didactic training; mixed effects have been observed with opinion leaders, audit and feedback. Interventions that have been generally effective are manual or computerized reminders, academic detailing, and multifaceted interventions [4,9]. Each approach presents specific challenges to implementation. The identification of local barriers to change represents a new challenge in the development of interventions adapted to each clinical environment [2,4].

Systematic reviews of studies of behaviour change have found that interventions are often not well described, or that effects from a particular method are difficult to evaluate [10]. There is inconsistent use of terminology, which contributes to difficulties in replicating and understanding the association between intervention and outcomes [10,11]. The studies are complicated by the fact that implementation is not something that happens at once; it can take several years to complete in many organizations [12].

Additionally, although a number of psychological theories and frameworks have been suggested in order to deepen our understanding of successful implementation, and to bridge the gap between clinical guidelines and practice, they are rarely used in studies in this area [13,14]. Fixen and colleagues [12] have developed a useful framework for implementation. Fixen's model of implementation makes a distinction between skills' transfer during training and implementation of the skills in practice. Effective implementation is achieved if core implementation components and core intervention components can be identified. The former are components for implementing the practice or programme and may include staff training, coaching, administrative structures and strategies, as well as policies to support the change. Core intervention components include programme theory, treatment components, programme structure and improvements.

Most of the studies focus on physicians' attitudes and barriers to the implementation of clinical guidelines. Only a few studies have examined barriers and facilitators experienced by other health care practitioners [15]. Among the few studies published concerning psychiatry, frequently reported barriers include lack of organizational support, clinicians' reluctance to change and concerns over the quality of the guidelines [16]. Further, the barriers include concerns about a "cook book" approach to medicine and oversimplification of complex clinical questions, lack of acceptance of guidelines' recommendations, practical barriers and a perceived challenge to the autonomy of the clinician. Effective facilitation strategies appear to emphasize the importance of effective feedback and multifaceted interventions [7]. Adaption to local circumstances has also been found to be valuable [17].

In order to extend knowledge about effective implementation strategies of clinical guidelines in psychiatric settings we performed an exploratory study. The aim of the study was to investigate perceptions of clinical guidelines and to identify barriers to, and facilitators for, their implementation.

More specifically, the following questions were addressed:

• What are practitioners' perceptions about implementing evidence in a psychiatric context?

• What factors do practitioners identify as the most important in enabling the implementation of clinical guidelines?

• Which factors do practitioners identify as hindering the implementation of new knowledge and clinical guidelines?

## Methods

As part of a larger programme evaluation we used a qualitative study design to explore the implementation of clinical guidelines in psychiatric care in Stockholm, Sweden.

### Implementation programme for clinical guidelines

In Stockholm County, representatives of public purchasers and providers meet on the Stockholm Medical Advisory Board in to order to develop clinical guidelines. The Stockholm Medical Advisory Board for Psychiatry has developed clinical guidelines for various psychiatric disorders. These guidelines have been developed to advise on the treatment, management and evaluation of psychiatric disorders. The guideline recommendations have been developed by multidisciplinary groups of health care professionals, researchers and purchasers. It is intended that the guidelines will be useful to professionals in psychiatric inpatient and outpatient settings as well as in primary care. The guidelines are intended to assist the interdisciplinary care team in the process of recognition, diagnosis, treatment (including pharmacotherapy, psychological therapy and psychosocial support), and monitoring.

After the publication of the clinical guidelines for depression in 2003, a pilot study was conducted in order to monitor implementation. An implementation programme was initiated and monitored by registering outcome and process quality parameters. Six psychiatric clinics participated. The guidelines were actively implemented at four clinics whereas two only received the guidelines and served as controls. A local multidisciplinary team was established at the intervention clinics. Implementation included seminars, regular feedback and academic detailing. The implementation team was led by an external psychiatrist, serving as facilitator. Facilitation was used as a model to challenge existing practice and support development and change. The role of the facilitator was to assist the health care providers in understanding what should be changed and how to achieve the desired results. One difference between facilitator and local opinion leader is that the facilitator uses interpersonal and group skills to attain changes, whereas an opinion leader's influence is primarily dependent upon status and competence [18,19]. A multifaceted intervention was used since the implementation programme involved two or more interventions targeting different barriers to change [4]. Academic detailing consisted of a trained person giving information to providers in their practice settings with the intent of changing their performance. Emphasis was put on a collaborative approach, critical reflection and changing practice culture. At each facility, a prospective identification of the barriers to change was carried out in order to define and adapt the intervention. Compliance to the guidelines was measured using quality indicators derived from the guidelines. In order to analyse the gap between clinical guidelines and current practice, an audit of medical records was conducted before, during and after implementation. These data could be used to design intervention strategies to reduce barriers and facilitate guideline implementation. Our previous studies showed sustained results at a two year follow-up [20,21].

### Participants

Two general psychiatric outpatient clinics providing care for people with depression were approached to take part in the present study. One participated in the active intervention; one only received the guidelines and served as a control. The two clinics were similar in their structure and organization.

Data were collected from a series of focus groups and individual interviews before and at the end of implementation in late 2004. All health care personnel in the implementation teams were asked to participate in the study. At the implementation clinic, all (100%) of the team members were interviewed; facilitator (*n *= 1), doctors (*n *= 4), nurses (*n *= 3), counsellor (*n *= 1), psychologists (*n *= 3), manager (*n *= 1), and the head of department (*n *= 1). Focus groups were conducted; two at the implementation clinic, one before and one six months after implementation, and one at the control clinic. The same participants took part in focus groups after the implementation. The focus groups before implementation were conducted to provide a broad perspective of factors that might be influential when implementing clinical guidelines. The focus group approach was used specifically to allow interaction between the participants on the questions raised. Participants react to and reflect on others' views, thereby, potentially leading to richer or deeper expressions of opinions or behaviour [22]. These data could be used to design future intervention strategies to remove system barriers and facilitate guideline implementation. At the control clinic, practitioners were invited to participate in a focus group in order to explore perceptions about clinical guidelines and how to translate evidence into practice in a psychiatric context. Focus group participants were: doctors (*n *= 5), nurses (*n *= 3), counsellors (*n *= 2), psychologists (*n *= 3) and a manager (*n *= 1). To further deepen our understanding we performed individual interviews guided by issues raised in the focus groups. Fourteen individual interviews were conducted before, and 14 six months after implementation at the intervention clinic.

The interviewees had a range of 4-31 years of psychiatric experience. The participants' ages ranged from 32 to 63 years. There were no detectable differences in responses according to practice size or gender. The age profile of both groups was similar.

### Interview procedure

Both the initial and follow-up interviews were semi-structured with open-ended questions and followed an interview guide. They took place at the practitioners' own offices. All focus groups and interviews were audio taped and transcribed verbatim by the interviewers directly after completion. The interviews were scheduled at the convenience of the participants. The focus group lasted approximately 90 minutes. The average length of each in-depth interview was 50 minutes.

The first author (TF) conducted the focus groups and a trained graduate research assistant conducted the individual interviews. Data collection was completed when it was deemed that a comprehensive picture of the implementation process and influencing factors had been attained. An interview guide was used for all focus groups and interviews. Facilitators and barriers to guideline implementation and adherence to guidelines were addressed.

The interview guide included the following themes:

• Trust in evidence and the guidelines

• How guidelines influenced the professionals

• What factors enabled implementation

• Barriers to using guidelines

### Data Analysis

The data were analysed using qualitative content analysis [23]. Both a manifest and latent content analysis were performed. In the manifest content analysis, the written words, directly expressed in the text were used for the analysis. In the latent content analysis, the aim was to find the underlying meaning in the text [24]. In the first stage of the analysis, the responses were read through line-by-line, in order to obtain an understanding of the text and overall impression of the material. Secondly, important meaning units (a word or a sentence) were identified and the texts were condensed. The data were further organized using the Open Code software, version 3.4 [25]. Thirdly, the meaning units were labelled with codes and grouped into categories and subcategories. Fourth, the codes, subcategories, and categories were continually refined and compared with each other [24]. During the analysis, the intention was to reduce the number of categories by aggregating similar categories into broader categories. Finally, the set of main categories was established by grouping together subcategories with similar meaning.

In analysing the data from the focus groups, we looked for differences and similarities in the health professionals' behaviour and perceptions, following the same procedure as for the interviews. Focus groups and in-depth interviews were analysed separately. Once all transcripts had been analysed, results were reviewed in order to describe findings that apply to the study as a whole. As the themes emerged, these were continuously validated against the data, by being compared to different pieces of actual text. The result were then discussed and revised together with an independent co-researcher (JH). Illustrative quotations were chosen from the interviews, as is standard practice in qualitative studies [26]. To ensure confidentiality all quotes from participants have been de-identified. Quotes with "I" indicate a member of staff from the intervention clinic and "C" a member of staff from the control clinic.

### Ethical considerations

All persons asked to be interviewed in the study agreed to participate. They were informed about the voluntary nature of their participation and their right to decline. Data are presented so that individual participants remain anonymous, and quotations used in any reports do not include information that could identify the participant. The study was approved by The Central Ethical Review Board at Karolinska Institutet, Sweden.

## Results

Three main categories were formed to describe barriers or facilitators for successful implementation of psychiatric clinical guidelines. Our analysis showed individual, organizational, and attitudinal factors related to perception of guidelines and strategies. These categories were: (1) organizational resources, (2) health care professionals' individual characteristics and (3) their perception of guidelines and implementation strategies. Table 1 uses these categories in presenting a summary of the barriers and facilitators influencing implementation of clinical guidelines as reported in the interviews.

**Table 1:** summarizes reported barriers and facilitators influencing implementation of clinical guidelines.

Categories and subcategories	Barriers	Facilitators
**Organizational resources**		
Staff	Lack of time	Clear roles
	No agreement on need to use clinical guidelines	Included in decision-making processes
	Emotional exhaustion	Sufficient time
	Influence of prior experiences	
	Workload	
	Information overload	
Learning culture	Lack of learning culture	Promotes learning organization
Leadership	A lack of dedicated time	Strong leadership
	Lack of investment from the organization	Active department chief
	Guidelines not mandatory	Head of department supported the implementation
	Lack of organizational strategy and skills	Effective organizational structures
	Resistance to multi-disciplinary team	Empowering approach to learning
	Concerns about resources	Multi-disciplinary implementation team
	Lack of financial resources	Awareness of clinic attitudes and actions
		Effective teamwork
Dissemination	Lack of clear intervention goals	Supporting implementation
	No regular implementation meetings	Planning the implementation process
	Guideline format	Access to guidelines tools and recommended clinical scales
Change clinical patterns	No measurement or tools for evaluation of care	Feedback on performance
		Audit used routinely
		Quality indicators
		Measuring 'before' in order to identify gap
Facilitation	Lack of facilitation	External facilitation
		Academic outreach visits
		Driving local change
Health care professionals' individual characteristics		
Attitudes and beliefs	Negative attitudes to clinical guidelines and new action	Positive attitudes and beliefs regarding guidelines and new action
	Perceived limited validity of guidelines	
	Fear of loss of autonomy	
	Fear of standardization of care	
	Concerns about relevance of evidence to own patients	
	Lack of internalization of guidelines	
Knowledge	Lack of research skills	Increased knowledge
	Lack of specialized training	
Perception of guidelines and implementation strategies		
Credibility of content	Change in recommendations	Increased accountability
	Overestimation of current care	
Awareness	Lack of familiarity with guidelines	Practitioner's awareness

The first column represents categories and subcategories. Examples of factors influencing the implementation work as reported in the interviews (columns 2 and 3).

### Organizational resources

Resources were raised as an essential issue that enables the progress of implementation work. There was general consensus among practitioners at the control clinic concerning lack of trust in the guidelines' recommendations and an environment not supportive to clinical guidelines was described. It was suspected that financial motives often lay behind clinical guidelines, and there were concerns that cost control and standardization of care might threaten the doctor or therapeutic-patient relationship. Loss of autonomy, and beliefs about standardized care were also described by the non-implementers. One clinician explained: *"I'm afraid that the clinical guidelines lead to a standardized care, we cannot meet the patients' needs...my long clinical experience is no longer valuable..." (C)*.

The health practitioners at the control clinic reflected on this perceived concern about losing control. One of the participants said:

*"...standardizing the content of the meeting with the patient and care, I see as very difficult" (C)*.

At the control clinic lack of time was highlighted as a barrier. However, this was not addressed by the interviewees at the implementation site. Time factors were characterized by the experience that there was inadequate time for training based on the guidelines, implementation into clinical practice, or updating the evidence from research literature.

*"We do not have time to read and take note of all the scientific treatment guidelines and relevant literature for our profession or field" (C)*.

One factor reported to be successful was an active leadership with senior administration supporting clinical guidelines. This served to increase awareness and willingness to change clinical practice Support from the local leader and at department level was deemed important. Academic detailing was also identified as a promoter. The expert-facilitated dialogue encouraged others to measure change, and promoted guideline acceptance within the implementation team.

*"...our implementation leader influenced the process by calling meetings, facilitating discussions, creating a positive atmosphere and encouraging the team to increase our knowledge" (I)*.

During the implementation and adaptation phase, good leadership and consistent communication was described as being fundamental to the successful implementation of guidelines. Participants described leadership support and an organizational vision emphasizing guideline implementation as facilitators. Concerns about lack of investment from the organization and lack of organizational strategies were identified as barriers. Participants at the control clinic felt that they did not have support from senior administration in implementing the guidelines or working according to their requirements. Practitioners felt they lacked authority to effect changes and were not certain how to implement the clinical guidelines in their practice in an effective and organized way. Thus practitioners from the control clinic were more pessimistic and felt constrained by resources and the organization.

The issues of creating a supporting environment and providing support for changing clinical patterns were addressed. Most of the participants described the difficult task of deviating from established practice patterns. Practitioners reported that a major barrier to using guidelines in practice was that they did not always have access to recommended diagnostic assessment tools and standardized rating scales. One practitioner said: *"I mean, how can you change your clinical practice when we don't have access to, or adequate skills to use, recommended tools?" (C)*.

To observe changes in clinician behaviour requires knowledge of the baseline care. Regular audits of patient care delivered by the clinicians were reported to be of help in identifying ongoing important gaps between current care and guideline recommendations. One of the practitioners said: *"At first, I thought it was very difficult... Then we started to get the hang of things, and really saw that we all were improving..." (I)*.

At the implementation clinic, audit data were used to inform the implementation teams about practice change. Quality indicators were collected as part of implementation intervention and used for learning and adjusting practice and services. After implementation, the participants in the focus groups expressed the importance of information gathering or auditing in order to access the gap between knowledge and clinical practice.

*"Indicators helped us to support the change and identified what needed to be improved... It was so obvious that we were not using some of the effective methods to any great extent; they also showed us that we were not putting some of the recommended methods into practice" (I)*.

*"Indicators from the guidelines gave us a clear picture of the gap between guidelines and practice. Gave me a clear overview of my own and colleagues' work... without audit and feedback we were not sure what we needed to change, and would not know if we're improving" (I)*.

A strong theme emerging from focus groups and interviews from the implementation site was the positive benefits of having a multidisciplinary implementation team. Participation in the team resulted in a sense of local ownership of the implementation and practice changes. It also gave team members an opportunity to consider the evidence involved.

*"Most probably its strength was that it was a multi-disciplinary team...We could see the results when other professions got involved in the care... It certainly changed my view of others' knowledge and capacity..." (I)*.

The emphasis on working across disciplines, identifying areas for a collaborative and team-oriented approach was seen as essential for successful local implementation. One example was that assessment using the standardized rating scale could be performed by other professionals than physicians.

Practitioners reported that the focus group sessions acted as a strong facilitating factor, and that they promoted knowledge and the implementation of guidelines.

Providers gave many example of ways in which guidelines helped them in their clinical practice; in clinical decision making, in setting treatment goals and in evaluating outcomes. Apart from direct patient encounters, the guidelines and the quality indicators stimulated quality improvement initiatives. In the implementation group, providers believed that using the guidelines would result in an improved quality of care.

### Health care professionals' individual characteristics

Participants who believed that implementation of clinical guidelines would result in improved outcomes for patients and a more effective care had a positive attitude towards implementation and the guidelines.

*"When we examined the psychiatric care that we gave the patients and considered outcome from the patient's point of view, this gave us an insight regarding our ability to describe the treatment, assess it and not least the opportunity to see if the patient recovered after our intervention" (I)*.

Lack of knowledge, skills and motivation were described as major barriers to implementation and the use of research findings in clinical practice. A failure to internalize guidelines into clinical routines was also identified as a barrier to guideline implementation. Participant perspectives on the barriers to using clinical guidelines in clinical work were identified. The need to bridge the gap between knowledge and skills was a perspective described by participants.

*"I know it's quite silly. I mean I know it's only a matter of starting to do it, but still we don't change our behaviour. ... I'm not sure that we have the skills... it's so hard to reflect upon our own and colleagues' behaviour" (I)*.

*"...The clinical guidelines really help us to understand that there is a gap between what we do and the evidence... It's clear what we are supposed to do... It's also fascinating to suddenly understand that there is a large gap between what we think we are doing and what we really do..." (I)*.

Guidelines were seen as necessary, but sometimes not an adequate aid to decision-making.

*"...We need to work more systematically and structured in our clinical work... It is a tradition in psychiatry to choose treatment and methods based on one's own clinical experience... There is a lack of support for people with psychiatric co-morbidity..." (I)*.

Barriers related specifically to psychiatry as a medical discipline were described and differences between psychiatric and other medical specialties were highlighted. Most participants thought that there was a definite difference in attitudes to, and knowledge about, the guidelines and how to practice evidence-based medicine in the psychiatric discipline compared to somatic specialties.

*"We have no tradition in psychiatry of following clinical guidelines. It is a new approach and requires great adaptation.... "(I)*.

The guidelines led to discussions between representatives of different schools of thought and theories in psychiatry. Traditional treatment approaches were questioned in the light of presented evidence and this was addressed as a barrier.

*"...difficult for me as a psychotherapist to possess knowledge and skills that do not comply with modern requirements. There are great demands to change my clinical work..." (C)*.

Several practitioners addressed the complexity of using evidence-based medicine in practice.

*"During my residency training at an internal medical department, no one contested the guidelines. It was a part of one's work to be guided by clinical guidelines, based on evidence. Quite differently, today, I feel resistance and that I am questioning a colleague if I bring up the issue of whether our treatments are based on evidence and guidelines" (C)*.

All practitioners had been exposed to research-based teaching. In the focus group there was a consensus that being taught about research enabled them to learn how to question, look for evidence and evaluate its relevance for practice. Learning new skills was initially experienced as increased workload and stress, but it led to a new conceptualization of the discipline and generated new practice-based knowledge.

*"...you seek the evidence and evaluate the evidence for practice, ... you don't rely on what others do..." (I)*.

The relationship between higher levels of qualification and research utilisation were addressed in the interviews. Further training led the providers to become more knowledgeable, confident and aware of the importance of research.

*"Further training has made me critically appraise the evidence for treatment and its validity and try to improve the quality and outcome of care. It makes you aware of the need to evaluate your methods and aware of the importance of research" (I)*.

Several providers felt that the guidelines were not presented in a user-friendly format, were too long, disorganized and difficult to access on-line.

### Perception of guidelines and implementation strategies

At the control clinic the participants said that they were unfamiliar with the published guidelines. The lack of familiarity was often attributed to the overwhelming amount of medical research, and difficulties in keeping up to date with recent recommendations.

A belief that the guidelines originated from unreliable sources as well as doubts about their authors' credibility were noted as barriers. 'Missing' recommendations or a lack of addressing issues believed to be important for clinical practice and for patients, influenced the providers' willingness to accept guidelines.

Participants expressed concern about the applicability of guidelines in their own clinical practice. Providers noted difficulties in applying guidelines to specific patients, in particular, patients with psychiatric comorbidities and the elderly. The difficulty of applying guideline recommendations, e.g. a standardized rating scale, to specific populations, in particular, non-Swedish and non-English speaking persons, was also noticed.

Providers typically overestimated the quality of current psychiatric care. Audit and feedback gave providers at the intervention clinics a meaningful insight into their own practice.

## Discussion

New methods in psychiatry, as in all other areas of medicine, are continuously introduced but implementing evidence to practice is complex and there is no simple solution [2,6,27]. Implementation and change of praxis are complicated processes involving individuals, teams and organisations. The purpose of using qualitative methods in this study was to gain a deeper understanding of barriers and facilitators for implementing clinical guidelines in psychiatry in a multidisciplinary team. An understanding of what influences practitioners' behaviour and whether and why clinicians use evidence in practice has gradually increased by contributions from qualitative research.

There were three main areas that differentiated the practitioners at the control clinic from those at the implementation clinic: (1) concerns about control over professional practice, (2) beliefs about evidence-based practice and (3) worries about underlying financial motives. In the focus group at the control clinic negative attitudes to guidelines in general and underlying concerns about financial motives emerged as key findings. The practitioners expressed less belief that clinical guidelines could be useful for their practice. They were also more concerned about their lack of control over implementation of the guidelines (lack of ownership), over their practice, and over their professional role (lack of autonomy). They perceived more negative effects, both for themselves and for the patients' care. These attitudes and barriers were not seen at the implementation clinic, where participation, encouragement and ownership issues were addressed. Financial motives were not addressed as a main barrier. The interviewees reflected on potentially successful strategies such as having a facilitator who helped them to address the gap between clinical guidelines and practice. Facilitation has previously been identified in the literature as a potentially important component in the implementation of research findings. However, the concept is not well-defined in this field and future research should address this issue [28]. Garbett and McCormack [29] have stated that practitioners need help in identifying organisational factors that impede progress, in order to achieve a greater sense of ownership and empowerment. This was seen in the interviews at the implementation clinic where auditing and information gathering were seen as an important contribution in supporting the local changes. Implementation requires an exploratory assessment of contextual issues. Knowledge about local barriers to using guidelines, providers' attitudes, beliefs and preferences have been identified as important for planning implementation strategies [5,17]. A high degree of ownership in the implementation process was also revealed, and this has previously been reported as an important factor in the utilization of guidelines and research [4,30,31].

The resource issue was addressed in the interviews, lack of resources as a barrier was mentioned both at the intervention and the control clinic. Interestingly, only the practitioners at the control clinic mentioned lack of time as a barrier. Limited time for research implementation is a frequently cited barrier in the literature [32]. The fact that this was not reported at the interviews at the intervention clinic might be due to the fact that the implementation clinic team tried to change and develop practice and did not experience lack of time. It has previously been reported that changes in practice cannot occur without an organized approach which most likely had occurred at the implementation clinic [33].

Organizational leadership was frequently discussed and might be the key to evaluating the needs of the organization, identifying the resources required, and creating a strategic plan for implementation. A supportive organizational culture and the presence of active leaders to guide the implementation and clinical changes were described as facilitators in the interviews. Leaders who failed to develop a practical vision of implementation and change and who were not involved themselves in the implementation process were described as barriers. Amongst participants who less actively supported the implementation of clinical guidelines, key barriers included lack of authoritative support to change and weak leadership. Limited support from colleagues, supervisors and organizations are frequently reported in the literature as negatively influencing guideline implementation [32]. Pettigrew et al. [34] have previously suggested that successful change is more likely to occur in contexts with a supportive organizational culture and leadership.

Overall, the interviewed health care professionals gave many examples of ways in which guidelines could help them in clinical decision-making. Most importantly, they believed that using clinical guidelines would result in an improved quality of care, and would eventually save lives. The presence of a multidisciplinary team was regarded as having a positive effect on implementation. This has also previously been proposed as essential to implementation [35]. In summary, we found that the practitioners at the implementation clinic had a positive attitude towards using the guidelines. They believed that using the clinical guidelines would result in a higher standard of care, and promote the use of evidence-based medicine. However, they were concerned that the guidelines would be of no help in patients with multiple psychiatric diagnoses.

The present study differs from others in that we interviewed all members of the multi-professional team at a psychiatric outpatient clinic, rather than only psychiatrist, were interviewed.

No particular barriers or facilitators were reported more often in any of the professions. Age, gender or previous length of experience did not seem to have an influence on the reports, which is consistent with previous studies [36-38].

Our study has several strengths. We report interviews from participants in a real-life implementation project that included a multi-faceted implementation strategy. In a previous paper we have reported on sustained compliance to the implementation of guideline recommendations over a two year period [20,21].

Even if a multi-professional team developed the implemented guidelines, the format may have influenced the practitioners' attitude [39]. The study was conducted in one part of Sweden and further research needs to be conducted in other settings to assess the extent to which our results are generally applicable.

Additionally, the results might have been influenced by the fact that the first author conducted the focus groups and was involved in planning and conducting academic detailing in the programme. Although analysis of the effectiveness of using academic detailing and evaluation was not the purpose of this study, their use was investigated by the research assistant in the individual interviews.

## Conclusions

Getting evidence into practice and implementing clinical guidelines are dependent upon more than practitioners' motivation. There are factors related to the local context - for example, culture and leadership, evaluation, feedback on performance and facilitation - that are likely to have an influence. There were three main areas that differentiated the practitioners at the control clinic from those at the implementation clinic: concerns about control over professional practice, beliefs about evidence-based practice and suspicions about underlying financial motives.

## Competing interests

The authors declare that they have no competing interests.

## Authors' contributions

TF, AÅW, MB and YF have all participated in the design of the study. TF, YF and JH have analyzed the data. TF drafted the manuscript and all other authors participated in a critical revision of the draft as well as contributing important intellectual content. All authors read and approved the final manuscript.

## Pre-publication history

The pre-publication history for this paper can be accessed here:

http://www.biomedcentral.com/1471-244X/10/8/prepub
